# Modeling Mastitis Risk Management Effects on Dairy Milk Yield and Global Warming Potential

**DOI:** 10.3390/ani15010050

**Published:** 2024-12-28

**Authors:** Giulia Ferronato, Anna Simonetto, Gianni Gilioli, Alfonso Zecconi

**Affiliations:** 1 Department of Civil Engineering, Architecture, Environment, Land Planning and Mathematics (DICATAM), University of Brescia, 25121 Brescia, Italy; anna.simonetto@unibs.it (A.S.); gianni.gilioli@unibs.it (G.G.); 2 One Health Unit, Department of Biomedical, Surgical and Dental Sciences, School of Medicine, University of Milan, 20133 Milan, Italy; alfonso.zecconi@unimi.it

**Keywords:** dairy, mastitis, risk assessment, global warming potential

## Abstract

Mastitis represents a significant challenge for dairy farming, resulting in economic losses and environmental impacts. This study assesses a model for the evaluation of the impact of mastitis on dairy productivity and Global Warming Potential (GWP) under diverse management scenarios. The model considers a range of factors, including bedding materials, milking systems, health surveillance, and overcrowding. The results of the simulation demonstrate that effective management, encompassing the utilization of sand bedding, and the presence of an annual herd health monitoring plan have the potential to reduce the prevalence of mastitis and enhance milk yield by up to 10% in milking parlors and 7% in automatic milking systems. At the herd level, the GWP ranged from 1.37 to 1.78 kg CO_2_eq/kg Fat- and Protein-Corrected Milk (FPCM), with the use of sand bedding resulting in a 14% reduction in GWP, while the utilization of non-composted manure-based materials led to an increase of 12%. The occurrence of overcrowding and a lack of adequate cleanliness in resting areas were found to have a markedly detrimental impact on both productivity and the environmental performance of cows. These findings illustrate the dual benefits of enhanced mastitis management, namely improved milk production and reduced environmental impact. They offer valuable insights for farmers and policymakers alike.

## Simple Summary

Mastitis is a common and costly disease in dairy cows, affecting milk production and animal welfare. It also increases the environmental impact of dairy farming by contributing to greenhouse gas emissions. This study aims to evaluate how different farm management practices affect the risk of mastitis and improve both milk production and environmental sustainability. This study presents a model for the evaluation of the impact of mastitis risk factors on dairy productivity and Global Warming Potential under diverse management scenarios. The model considers a range of factors, including bedding materials, milking systems, health surveillance, and overcrowding. The models estimate that the use of sand bedding and continuous health checks significantly reduced the incidence of mastitis and increased milk yield, thus reducing the environmental footprint of the farm. On the other hand, overcrowding and poor hygiene in resting areas increased both the risk of mastitis and the environmental impact. This research highlights the importance of good farm management practices to improve cow health, increase productivity, and reduce environmental impact. These findings can help farmers adopt more sustainable practices while maintaining or increasing milk production, benefiting both the industry and the environment.

## 1. Introduction

Mastitis represents one of the most prevalent and costly health problems in dairy farming. It has a considerable impact on cow welfare, resulting in a reduction in milk yield and a deterioration in hygiene (i.e., colony forming units, somatic cell counts) and quality [1]. At the farm level, these issues result in economic losses due to decreased milk production, increased discarded milk, reduction of milk quality-based payment, higher antimicrobial usage, and potentially elevated cow culling rates [2,3,4,5].

An effective control of mastitis should include the detection of mastitis-causing pathogens, and it is based on the identification and reduction of risk factors associated with the host characteristics (age, physiological stage, nutritional, and immune status) and environmental conditions [2,3]. Environmental factors encompass barn management, milking systems, feeding practices, and farm biosecurity [4]. Poor barn management, such as inadequate bedding, overcrowding, and insufficient health monitoring, can significantly increase the risk of mastitis by raising bacterial contamination of teats and reducing cow welfare [5,6,7].

According to the National Mastitis Council (NMC), mastitis-causing pathogens are generally classified into three groups: opportunistic, environmental, and contagious [8,9]. Environmental and contagious mastitis have a major impact on animal health and productivity. Contagious mastitis is primarily transmitted from cow to cow during milking, with the most common pathogens being *Staphylococcus aureus*, *Streptococcus agalactiae*, and *Mycoplasma bovis* [10]. In the event of the presence of contagious bacteria, this can lead to the animal acting as a reservoir [11,12]. In contrast, environmental mastitis is associated with pathogens present within the barn environment, including *Escherichia coli* and coliforms, *Streptococcus uberis,* and other environmental streptococci [10]. This is frequently the result of inadequate management practices and unsuitable housing conditions [13]. The failure to promptly identify subclinical forms can consequently result in a prolonged deterioration of the animal’s health status.

Mastitis has a detrimental impact on animal health and productivity while also contributing to an increase in environmental impacts. The inflammatory condition results in a reduction in milk production and an overall decrease in efficiency among affected animals, leading to an increase in the greenhouse gas (GHG) emissions and global warming potential (GWP) of 1 kg of dairy milk [14]. The inflammatory status may result in modified animal feeding behavior, which could lead to increased enteric emissions and organic matter excretions, with subsequent consequences for manure emissions [15]. Furthermore, mastitis may lead to increased culling and replacement rates. Farm management strategies, such as bedding material and milking system energy consumption, also influence the GWP.

Several authors investigated the mastitis impact on GWP, which ranged from 2.5% to 10% per unit of milk [16,17,18,19]. On a broader scale, the impact of mastitis extends beyond individual farms. For example, Guzmán–Luna et al. (2022) reported milk production losses in the European Mediterranean basin ranging from 0.37% to 3.21% [20,21]. This highlights the importance of reducing disease and improving animal welfare as strategies for achieving GHG reduction targets [22].

The importance of addressing risk factors associated with mastitis cannot be overstated, as it plays a pivotal role in both animal welfare and environmental sustainability. The development of predictive models to assess the incidence of mastitis, considering the influence of multiple factors and its impact on the environment, is becoming increasingly important in order to identify the most effective mitigation strategies. The current statistical approaches permit the modelling of mastitis at different levels (individual cow or herd) and for various objectives [23]. The primary focus of the available models concerns the qualitative and quantitative impact of mastitis on milk [24,25,26], the identification of the current event [27,28], the incidence [29], and the risk of infection and tolerance among the dairy herd [30]. To date, only Mostert et al. [17] have proposed a simulation model to detect GHG variations associated with the mastitis event. However, to the best of our knowledge, no one has yet proposed a model for assessing the risk of mastitis incidence in terms of environmental impact.

The aim of this paper is to present a model to evaluate how mastitis and the main risk factors associated with animal management strategies and conditions influence the sustainability of milk production. The model evaluates the impact of mastitis, together with factors such as management of resting areas, milking systems, and hygiene practices on milk production and its environmental sustainability. A simulation study was conducted to evaluate different animal management strategies and hygienic conditions. For each scenario, the impact on mastitis risk, milk production, and GWP has been estimated.

## 2. Materials and Methods

The model evaluates the impact of different animal management strategies and conditions on the GWP value of milk, considering the incidence of environmental and contagious mastitis under different scenarios.

**Figure 1:**
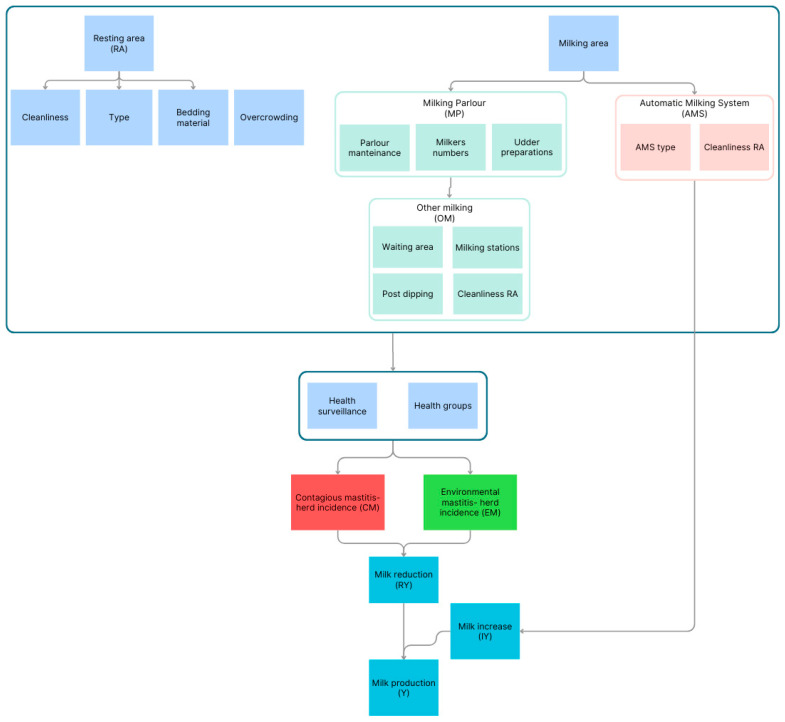
Flow chart of model to assess the mastitis risk incidence and expected milk yield.

The expected milk yield production (kg/day; *Y*) was assessed considering: a baseline milk production ($Y_{da}$), the increase in milk production (*IY*) due to automatic milking system (*AMS*), the reduction in milk production (*RY*) depending on welfare status, the proportion of herd animals affected by contagious mastitis (*CM*), and environmental mastitis (*EM*) (Figure 1).

The coefficients were set in accordance with the Italian production scenario, which is characterized by hot and humid summers and relatively cold winters. The parameters were set for Holstein cows reared in loose housing conditions on permanent bedding or bunk systems with restricted access to outdoor areas. All the model parameters were estimated mainly from the available scientific evidence, as reported in the references or by authors’ personal estimation from data obtained during extension service activity, and they are described in Supplementary Tables S1–S6 [8,21,31,32,33,34,35,36,37].

The expected milk yield production (*Y*), the expected milk yield increase (IY), and the expected milk yield reduction (RY) are estimated as (parameters are described in Table S1):

(1) $$Y=\left(IY-RY-\alpha_{C}\times CM-\alpha_{E}\times EM\right)\times Y_{da}$$

(2) $$IY=\beta_{YP}\times I_{YP}\times {IY}_{MA}$$

(3) $$RY=\rho_{RY}\times (\min\left({RY}_{RA}+{RY}_{MA}+{RY}_{OM};1\right))$$

The proportion of contagious mastitis (*CM*) is assessed differently if health groups are absent (Equation (4)) or if separated groups are present in the farm (Equation (5)):

(4) $$CM=\gamma_{CM}$$

(5) $$CM=\gamma_{CM}\times (\omega_{RA}\times {CM}_{RA}+\omega_{MA}\times {CM}_{MA}+\omega_{OM}\times {CM}_{OM}-I_{MM})$$

With regard to the incidence of environmental mastitis (*EM*) among the herd, this was calculated as:

(6) $$EM=\delta_{EM}\times \left(\omega_{RA}\times {EM}_{RA}+\omega_{MA}\times {EM}_{MA}+\omega_{OM}\times {EM}_{OM}-I_{MM}\right)$$

The weight parameters of *CM* and *EM* are reported into Table S2.

The values of IY*,* RY, CM, and EM depend on the management strategies and hygienic conditions of resting area (RA) and milking processes (Milking area—*MA*), as well as other milking system characteristics (*OM*). Details on the parameters related to these aspects are reported in Tables S3 and S4.

### 2.1. Resting Area (RA)


(7) $${RY}_{RA}=\rho_{OC}\times I_{OC}+\rho_{DR}\times I_{DR}$$

(8) $${CM}_{RA}=\gamma_{OC}\times I_{OC}+\gamma_{DR}\times I_{DR}$$

(9) $${EM}_{RA}=\delta_{OC}\times I_{OC}+\delta_{DR}\times I_{DR}+\delta_{MT}$$

### 2.2. Milking Area (MA) and Other Milking System Characteristics (OM)

The model considers two components related to milking processes. The MA component focuses primarily on the brand of the automatic milking system (AMS) (if present), the number of milkers, the udder preparation, and the maintenance. The OM component considers the post-dipping, the waiting area, and the number of milking stalls.

Both MA and OM are assessed differently if there is an automatic milking system (AMS) or a milking parlor (MP).

For the AMS scenarios, four different types of AMS were considered according to Milanesi et al. [34]. The MA and the OM components were assessed as follows (see Table S1 for parameters details):

(10) $${IY}_{MA}=1$$

(11) $${RY}_{MA}=0$$

(12) $${CM}_{MA}=\gamma_{R1}\times I_{R1}+\gamma_{R2}\times I_{R2}+\gamma_{R3}\times I_{R3}$$

(13) $${EM}_{MA}=\delta_{R1}\times I_{R1}+\delta_{R2}\times I_{R2}+\delta_{R3}\times I_{R3}$$

(14) $${RY}_{OM}=0$$

(15) $${CM}_{OM}=0$$

(16) $${EM}_{OM}=0$$

For the MP scenarios, the MA and the OM components were assessed as follows (see Tables S1 and S5 for parameters details):

(17) $${IY}_{MA}=0$$

(18) $${RY}_{MA}=\rho_{MP}$$

(19) $${CM}_{MA}=\gamma_{MP}$$

(20) $${EM}_{MA}=\delta_{MP}$$

(21) $${RY}_{OM}=\rho_{PN}\times I_{PN}\times I_{DR}+\rho_{WN}\times I_{WN}+\rho_{MN}\times I_{MN}$$

(22) $${CM}_{OM}=\gamma_{PN}\times I_{PN}$$

(23) $${EM}_{OM}=\delta_{PN}\times I_{PN}+\delta_{DR}\times I_{DR}$$

### 2.3. Global Warming Potential (GWP)

The impact of mastitis on the GWP of milk production was assessed using the life cycle assessment (LCA) method [38]. A “cradle-to-farm-gate” approach was employed, incorporating all processes within the system boundaries related to farm activities such as feeding, electricity consumption, bedding materials, enteric methane emissions, and manure storage emissions associated with the breeding of lactating cows. Inputs and emissions related to the breeding of calves and heifers were excluded.

Depending on the simulation scenario, litter supply, slurry and manure proportion, and electricity consumption varied. The system assumptions included a cow body weight of 650 kg, a baseline daily milk production of 35 kg with average protein (3.35%) and fat (3.86%) content [39], diet crude protein of 16%, and slurry storage with a natural crust and without cover for manure. The Fat- and Protein-Corrected Milk (FPCM) production of the scenario was calculated considering the base milk production, plus the increase due to no change in fat and protein content and the production of animals with mastitis, both contagious and environmental. A reduction in fat (3.82%) and protein (3.32%) content was also considered [40]. The DMI was assessed according to the Intergovernmental Panel on Climate Change (IPCC) Tier 2 [41] DMI estimation [39] considering the baseline MY and milk fat content. To evaluate the diet GWP, no distinction was made between purchased and self-produced feedstuffs, but a representative emission factor of diets used in the Po Valley based on maize silage was used (0.65 kg CO_2_eq/kg DMI) [42]. Enteric methane emissions (EE) were estimates with IPCC [41] then converted into CO_2eq_ assuming an emission factor of 29.8 [43]. The EE were assessed assuming a DE of 70% and NDF of 35% and Ym equal to 5.7. The characteristics in terms of the composition of the different bedding materials, the daily supply of materials, and emission factor for the different types of resting areas are shown in Supplementary Table S6. The relative emission factor was considered from Ecoinvet and AgriFootprint database. In order to define the amount of slurry and manure produced by the scenarios, the reference amounts according to the Directive 91/676/EEC [37] were considered. Volatile solid (VS) and nitrogen (Nex) excretions were assessed with IPCC [28]. The CH_4_ and N_2_O (direct and indirect) emissions were assessed with IPCC [28], assuming a slurry storage with natural crust and manure storage without cover. The energy consumption for AMS and MP was assessed equal to 0.021 kWh/kg milk for MP and 0.0612 for AMS [44]. For the emission factor, the average Italian grid mix was considered (0.525 kg CO_2_eq/kWh) [45].

The overall GWP (kg CO_2_eq/kg FPCM) was assessed considering the enteric methane emissions (EE), feed consumption (FC), bedding material (BM), energy consumption (EN) and manure storage emissions (ME):

(24) $$GWP=\frac{{CF_{EE}+{CF}_{FC}+CF}_{BM}+{CF}_{EN}+{CF}_{ME}}{Y}$$

### 2.4. Scenario Analysis

The model enables the evaluation of the effects of different animal management strategies and barn hygiene conditions on the risk of mastitis, along with the corresponding impact on milk production and GWP. To conduct this assessment, a simulation study was developed based on scenarios generated by varying the conditions considered within the model. These scenarios represent a factorial combination (when pertinent) of the following variables: resting area (type, cleanliness, materials, and level of overcrowding), milking system (AMS or MP), number of milkers and stalls, udder preparation, maintenance, post-dipping, waiting area, presence of health groups, and status of milk monitoring.

### 2.5. Statistical Analysis

Data were analyzed using R software (R Core Team, 2024; version 4.4.0). Upon determining non-data normality, Kruskall Wallis rank sum tests (package stats) were used to test the existence of significant impact of the parameter on milk yield and milk GWP.

## 3. Results

According to the variable defined, 27,456 scenarios with a milking parlor (MP) and 1152 scenarios with the automatic milking system (AMS) were developed. The reported results are based on the assumption that the herd is always infected with contagious pathogens causing mastitis.

### 3.1. Milking Parlor

The main results of scenarios with MP are reported in Table 1. The mean FPCM herd production of the overall scenarios was 29.99 ± 1.96 kg, representing a 13% reduction in comparison to the baseline scenario. All model parameters had a significant effect (*p* < 0.001) on the change in FPCM production and GWP of 1 kg FPCM and on the contribution to GWP of the enteric emissions, feed, and manure emissions clusters (*p* < 0.001).

The factors that exerted the greatest influence on milk production were identified in overcrowding, health surveillance, the management of health groups, the cleanliness of the resting area, and post-dipping management.

In scenarios involving overcrowding, milk production decreases on average by 16.26%, which is 6.2% greater than in comparable scenarios without overcrowding. The implementation of continuous health surveillance for the detection of contagious bacteria has been shown to result in a reduction of milk loss by 10% compared to scenarios without continuous monitoring, where the loss can be as high as 16%. The type of resting area significantly influences milk yield reduction, with cubicle systems showing comparatively lower reductions than deep litter systems. Additionally, maintaining cleanliness in both cubicles and deep litter areas can mitigate milk yield reduction by up to 15%. Among litter types, sand and straw were associated with smaller reductions in production, with sand yielding only a 12% reduction. In contrast, separated solid non-composted digestate showed a higher reduction at 14%.

The adequacy of post-dipping routine and udder preparation during the pre-dipping phase were identified as the milking parlor practices with the most substantial impact on milk production. Improper pre-dipping routines can reduce production by up to 14%, while inadequate post-dipping practices may lead to losses of up to 15%. Additionally, the presence of a health group and continuous health surveillance significantly reduced the spread of contagious mastitis. Implementing a surveillance program also lowered the incidence of environmental mastitis within the herd.

The GWP of the MP scenarios exhibited a range of 1.37 to 1.78 kg CO_2_eq/kg FPCM, with an average value of 1.59 kg CO_2_eq/kg FPCM. Manure storage contributed the most to GWP (50%), followed by enteric methane emissions (23%) and feeding practices (23%).

Regarding the influence of risk factors on GWP variation, the use of sand as bedding material led to the most significant environmental impact reduction, lowering the impact by 14% (1.37 kg CO_2_eq/kg FPCM).

The type of resting area affected greenhouse gas emissions, with cubicle-based systems showing a potential 2% reduction in GWP compared to deep litter systems. Conversely, using bedding materials made from non-composted manure increased GWP by 12%, reaching 1.78 kg CO_2_eq/kg FPCM. The absence of overcrowding was shown to reduce GWP by 8%. Additionally, maintaining a high level of cleanliness in the resting area can further lower GWP to 1.52 kg CO_2_eq/kg FPCM, representing a 7% reduction.

The implementation of health management strategies, specifically group management and continuous surveillance, demonstrated a potential reduction of 2% in cases where a separate group is involved, and surveillance is maintained on a consistent basis. Proper management of two specific milking parlor processes—udder preparation and post-dipping—showed the greatest potential for reduction in GWP, by 1.5% and 1.7%, respectively.

To optimize both production and GWP, the best performance is achieved by managing separate groups with continuous monitoring, utilizing sand as bedding material, and implementing appropriate pre- and post-dipping routines. In deep litter scenarios, this results in 34.16 kg FPCM and 1.07 kg CO_2_eq/kg FPCM, while cubicle scenarios yield the same production but slightly higher GWP at 1.14 kg CO_2_eq/kg FPCM. Conversely, the poorest performance occurs in scenarios lacking these characteristics and using non-composted manure-based materials, resulting in 25.28 kg FPCM and 2.26 kg CO_2_eq/kg FPCM.

### 3.2. Automatic Milking System

The main results of AMS scenarios are reported into Table 2. The average daily mean FPCM production in the AMS was 34.75 ± 4.26 kg.

All model parameters had a significant effect on the change in FPCM production (*p* < 0.05 for the type of resting area and bedding *p* < 0.001 for all other parameters) and GWP of 1 kg FPCM (*p* < 0.001), as well as on the contribution to GWP of the enteric emissions, feed, energy consumption, and manure emission clusters. The bedding material contribution variation was affected only by the resting area type (*p* < 0.001), cleanliness (*p* < 0.05), and bedding material type (*p* < 0.001).

Milk yield was more influenced by the presence of continuous health monitoring, followed by the cleanliness of the resting area (+6%). The type of resting area did not show a strong effect, but the presence of overcrowding could reduce the milk yield by about 5%. The presence of health groups, reducing the number of cows affected by contagious mastitis, improved performance by 4.68%. As reported for the MP scenarios, continuous health surveillance could improve by about 7%, reducing both the cows with contagious and environmental mastitis. The type of AMS used can also play an important role. Type A can increase performance by up to 4%, while Type C can decrease it by 4%.

The mean GWP value was equal to 1.43 ± 0.26 kg CO_2_eq/kg FPCM. The greatest contribution to the GWP value was that of manure storage (50%), followed by enteric methane emissions (27%) and feeding (23%). The bedding material influenced both the milk production greatly, but also the GWP variations due to the double effects on production and environmental costs of the materials. In the sand scenarios, the GWP achieves values of 1.23 ± 0.18 kg CO_2_eq/kg FPCM. Meanwhile, the solid-separated digestate not composted achieved values of 1.60 ± 0.25 kg CO_2_eq/kg FPCM. The cleanliness of the resting area and the absence of overcrowding could reduce the GWP to 1.31 kg CO_2_eq/kg FPCM (−8%).

The best performance of the AMS scenarios was identified under conditions of no overcrowding and adequate cleaning of the resting area with sand, together with management, allowing for the separation of health groups and a Type A AMS (deep litter scenarios: 43.12 kg FPCM and 0.93 kg CO_2_eq/kg FPCM; cubicle scenarios: 43.10 kg FPCM and 0.98 kg CO_2_eq/kg FPCM).

## 4. Discussion

The scenario analysis developed by applying the mastitis risk model allowed for the identification of the most effective animal management practices to optimize productivity and reduce greenhouse gas emissions.

The results clearly demonstrate the negative effect of mastitis on milk yield in both MP and AMS scenarios. Mean daily production for the MP system was significantly reduced by mastitis to 29.99 ± 1.96 kg, corresponding to a 13% reduction compared to baseline scenarios. Meanwhile, the AMS system showed a performance comparable to the production baseline scenario (34.75 ± 4.26 kg). The incidence of mastitis was still a significant threat to herd productivity; in fact, according to the bibliography, a mastitis event can result in a milk loss of 155–734 kg per lactation [5,41,42,43].

The risk model developed highlights that continuous health monitoring is a crucial intervention to mitigate the effects of mastitis. In both systems, continuous monitoring reduced the incidence of contagious mastitis, resulting in milk yield improvements of 7% in the AMS scenarios and up to 10% in the MP scenarios. The risk of environmental mastitis was also reduced in the AMS scenario, providing further evidence of the dual benefits of proactive herd health management. This highlights the importance of integrating regular health monitoring into dairy farms to limit the spread of infection and subsequent production losses. It is well known that somatic cell count (SCC) is a key indicator for the detection of subclinical mastitis. However, this only detects the presence of mastitis when the inflammatory process and the reduction in milk quality and quantity already started. Preventive screening for the detection of contagious agents is essential, along with procedures to minimize the risk of spreading infection. If not identified early, contagious bacteria can spread within the herd during milking, as hands, towels, and milking machines can serve as vectors for these pathogens [8].

Regarding the monitoring of environmental bacteria, inadequate management of resting areas can lead to heightened exposure to these bacteria. When coupled with ineffective pre- and post-dipping practices, this significantly raises the risk of udder contamination [46,47,48]. This underscores the importance of cleanliness in the resting area as a critical factor in preventing milk losses due to mastitis. A cleaner environment can mitigate production losses in MP and enhance yields in AMS.

Another significant finding of the study was the role of overcrowding and rest area conditions on mastitis incidence. Overcrowding not only increased the spread of contagious pathogens but also led to a significant reduction in milk yield of 16% in MP and 5% in AMS scenarios. The results of our model confirm that the adequacy of the resting area is critical in terms of comfort, management, and space [35,49]. An increase in animals per area, combined with a reduction in space and inappropriate cleaning, can lead to animals using unsuitable resting areas and udder contamination [50,51,52,53]. This suggests that proper environmental design and management are essential to ensure animal welfare, which, in turn, supports optimal milk production and reduces the risk of health problems.

In the specific case of the MP scenarios, their technical specifications also contribute to reducing impacts, albeit to a lesser extent than the previously mentioned factors. Optimal milking efficiency is achieved when the number of milking units aligns with the operator’s work routine and the cow’s milk production level [54,55]. This alignment minimizes both over-milking and operator downtime between the time the cow is ready for milking and the availability of the milking unit. It also results in proper udder preparation, which avoids bi-modal milk ejection [56,57]. In addition, the type of AMS used also influences the risk model, with Type A systems improving performance by up to 4%, while Type C systems showed a reduction in performance by the same margin. As reported by Milanesi et al. [34], despite AMS standardizing teat preparation and stimulation, there is a high frequency of two major mastitis risk factors: bimodality and irregular vacuum fluctuations. Furthermore, several authors showed that udder hygiene is crucial to minimize the risk of mastitis when AMS is applied [58,59].

Although mastitis is an inflammatory condition of the mammary gland that negatively affects the metabolic status and performance of animals, it is considered a disease with a moderate impact on greenhouse gas emissions. However, the impact on the entire supply chain should not be overlooked [16,60]. Several authors have reported an increase in energy losses through enteric methane emissions, with values ranging from 2.8 to 8% per kilogram of milk [18,19,20,21,22,23,24,25,26,27,28,29,30,31,32,33,34,35,36,37,38,39,40,41,42,43,44,45,46,47,48,49,50,51,52,53,54,55,56,57,58,59,60,61,62,63]. This results in an elevated milk unit GWP, reaching 6.2% for cows with clinical mastitis [17]. The contribution of the data cluster to the GWP values is in line with those reported by several authors for the same production system [42,64,65,66]. The GWP (kg CO_2_eq/kg FPCM) was found to be significantly influenced by the factors that increased the risk of mastitis. In the MP scenarios, it was found to be 1.59 kg CO_2_eq/kg FPCM, while in AMS scenarios, the mean value was slightly lower at 1.43 ± 0.26 kg CO_2_eq/kg FPCM.

The mastitis-risk model facilitates the assessment of differences in the impact of bedding materials and AMS, allowing informed decisions on potential investments to meet both production and environmental performance targets. Bedding materials can be broadly divided into two main categories: inorganic and organic. The organic category is further subdivided into non-manure-based materials and those derived from manure [35,49]. The physical, biochemical, and nutritional characteristics of the bedding directly influence bacterial growth [67,68].

The model confirms that bedding material played a crucial role in modulating GWP, with sand bedding being particularly effective in both reducing environmental impact (1.23 kg CO_2_eq/kg FPCM in AMS scenarios) and minimizing mastitis-related milk yield losses. In contrast, manure-based materials were associated with a higher milk GWP (1.60 kg CO_2_eq/kg FPCM). Manure-based bedding has no environmental cost because it is self-produced, but if not properly managed, it has the greatest risk of causing mastitis problems and, therefore, negative effects on production and GWP.

The type of resting area also affects effluent emissions by changing the proportion of slurry and manure produced, which vary in composition and physical characteristics, influencing the processes of nitrogen immobilization and fermentation of the organic component [69]. The type of bedding could reduce the contribution of bedding by 100% in scenarios with separated solid digestate or greatly increase it in scenarios with straw. Additionally, the type of bedding influences the contribution of the effluent emission cluster, as manure and slurry have different emission potentials and the nitrogen content in the effluent varies [70].

Proper management of resting areas (cleanliness and space availability) led to an 8% reduction in GWP in both MP and AMS scenarios, reinforcing the idea that complying with animal welfare requirements is the first strategy for mitigating impacts [71,72,73,74].

In order to comply with the requisite standards of animal welfare, it is of equal importance to implement appropriate protocols for the management of animal health. This is necessary both to prevent the spread of mastitis within the herd and to prevent the disease itself. For example, the model can predict that in scenarios where health groups are implemented and continuous health surveillance is maintained, the incidence of contagious mastitis can be significantly reduced, improving milk production by 58% in AMS systems [75].

The best-performing scenarios identified by the model occur under conditions of no overcrowding, adequate cleanliness of the resting area (preferably with sand bedding), and appropriate health management strategies, such as the use of health groups and continuous monitoring. These scenarios achieved the highest milk production (43.12 kg FPCM in AMS) and the lowest GWP (0.93 kg CO_2_eq/kg FPCM in AMS). In contrast, the worst-case scenarios, using non-composted manure-based bedding materials and without health monitoring protocols, resulted in lower production and higher GWP. The proposed GWP range is in line with what is found in the literature. However, this study always considered the presence of contagious mastitis. Further studies are needed to validate the model under field conditions representative of the production scenarios for which it was designed. In addition, adaptation of the model to different farming systems and production conditions is essential to improve its applicability. These improvements would allow the model to better reflect different management practices and environmental contexts. Exploring scenarios without the presence of contagious pathogens could yield even better results. In addition, assessing the impact of factors considering different productivity levels and cattle breeds would provide deeper insights into the relationship between animal welfare and environmental sustainability in different contexts.

This study emphasizes the interconnection between mastitis management, milk production, and environmental sustainability. Overall, the results indicate that reducing the risk of mastitis offers a dual benefit: it not only helps maintain or enhance production levels but also diminishes environmental impact. It is evident that the reduction in GWP is primarily influenced by changes in milk production. However, various factors can affect specific datasets.

The comparative analysis of MP and AMS scenarios reveals differences in how these systems respond to factors such as herd health management, overcrowding, resting area conditions, and bedding material, which collectively influence milk yield and greenhouse gas emissions.

The model can be used to assess the impact of decisions related to barn structure and farm management on mastitis risk, milk production, and environmental outcomes. It can serve as a tool in decision-support systems, either for evaluating decisions made during the planning phase of farm construction or for assessing the impact of changes to existing systems or protocols. This would help identify the most effective, farm-specific strategies.

## 5. Conclusions

The developed risk model provides a practical tool for the evaluation of mastitis-related risks, enabling farmers to make informed decisions that improve both animal welfare and environmental outcomes. Factors such as health monitoring, resting area management, and bedding practices are of significant importance in the reduction of mastitis incidence, which in turn leads to greater production efficiency and a reduced environmental footprint. A key conclusion is that enhancements in animal welfare must also be viewed in light of their influence on environmental performance. Nevertheless, further research is required to gain a fuller understanding of these trade-offs and to quantify them more accurately in order to provide a more comprehensive basis for sustainable decision-making in dairy farming. In light of these findings, the development of a decision-support system that enables the simultaneous evaluation of welfare conditions and environmental impact is necessary. Such a system would facilitate data-driven, balanced decision-making by farmers, thereby supporting both animal health and environmental sustainability and thus contributing to the development of a more resilient and sustainable dairy industry.

## Figures and Tables

**Table 1:** Effect of risk factors in milking parlor scenarios on milk yield, evaluated as protein- and fat-corrected milk (FPCM), incidence of contagious and environmental mastitis in the herd, and global warming potential (GWP; kg CO_2_eq/kg FPCM). The delta (∆) of GWP, enteric methane emissions, feeding consumption, bedding materials, energy consumption, and manure storage emissions were computed with respect to the mean value of all the scenarios.

Milking Parlor (MP)		FPCM ^1^	FPCM		CM ^2^ Herd	EM ^3^ Herd	GWP ^4^	GWP		Enteric Methane Emissions		Feed Consumption		Bedding Materials		Energy Consumption		Manure STORAGE	
		Scenario			Incidence	Incidence													
		kg	∆	*p*-Value	%	%	kg CO_2_eq/kg FPCM	∆	*p*- Value	∆	*p*- Value	∆	*p*- Value	∆	*p*- Value	∆	*p*-Value	∆	*p*- Value
Resting area	Deep litter	29.91 ± 2.00	−13.37%	<0.001	19.76% ± 0.12%	8.39% ± 0.09%	1.63 ± 0.29	2.31%	<0.001	0.11%	<0.001	0.30%	<0.001	48.46%	<0.001	0.00%	<0.001	3.88%	<0.001
	Cubicle	30.07 ± 1.93	−12.91%		19.69% ± 0.12%	6.86% ± 0.08%	1.55 ± 0.19	−2.35%		−0.11%		−0.30%		−49.14%		0.00%		−3.94%	
Overcrowding	Absent	31.05 ± 1.72	−10.06%	<0.001	18.70% ± 0.12%	6.54% ± 0.08%	1.47 ± 0.22	−7.46%	<0.001	−1.33%	<0.001	−3.56%	<0.001	−3.89%	<0.001	−0.01%	<0.001	−12.48%	<0.001
	Presence	28.91 ± 1.57	−16.26%		20.77% ± 0.11%	8.74% ± 0.09%	1.71 ± 0.22	7.56%		1.35%		3.61%		3.95%		0.01%		12.65%	
Resting area cleanliness	Clean	30.71 ± 1.90	−11.06%	<0.001	18.70% ± 0.12%	6.54% ± 0.08%	1.52 ± 0.24	−4.51%	<0.001	−0.89%	<0.001	−2.41%	<0.001	−3.31%	<0.001	−0.01%	<0.001	−7.38%	<0.001
	Not clean	29.26 ± 1.75	−15.26%		20.77% ± 0.11%	8.74% ± 0.09%	1.66 ± 0.24	4.58%		0.91%		2.44%		3.36%		0.01%		7.48%	
Bedding material	Sand	30.16 ± 1.89	−12.64%	<0.001	19.73% ± 0.12%	5.55% ± 0.07%	1.37 ± 0.16	−13.72%	<0.001	−0.23%	<0.001	−0.65%	<0.001	−74.15%	<0.001	−0.01%	<0.001	−26.04%	<0.001
	Straw, sawdust	30.09 ± 1.92	−12.85%		19.73% ± 0.12%	6.36% ± 0.07%	1.47 ± 0.16	−7.32%		−0.13%		−0.38%		142.67%		0.00%		−15.85%	
	Composted manure-based materials	29.89 ± 2.00	−13.43%		19.73% ± 0.12%	8.68% ± 0.09%	1.77 ± 0.22	11.45%		0.13%		0.37%		−100.00%		0.00%		23.60%	
	Manure-based materials with straw	29.98 ± 1.97	−13.18%		19.73% ± 0.12%	7.98% ± 0.08%	1.69 ± 0.19	6.29%		0.02%		0.05%		0.28%		0.00%		12.45%	
	Not composted manure-based materials	29.80 ± 2.03	−13.69%		19.73% ± 0.12%	10.04% ± 0.09%	1.78 ± 0.22	11.82%		0.25%		0.72%		−100.00%		0.01%		24.10%	
	Other materials	30.02 ± 1.95	−13.07%		19.73% ± 0.12%	7.25% ± 0.08%	1.46 ± 0.16	−8.13%		−0.03%		−0.10%		129.13%		0.00%		−17.49%	
Waiting area	Suitable	30.06 ± 1.98	−12.96%	<0.001	19.73% ± 0.12%	7.63% ± 0.08%	1.58 ± 0.25	−0.48%	<0.001	−0.08%	<0.001	−0.21%	<0.001	−0.21%	0.62	0.00%	0.493	−0.82%	<0.001
	Not suitable	29.93 ± 1.94	−13.33%		19.73% ± 0.12%	7.63% ± 0.08%	1.60 ± 0.25	0.48%		0.08%		0.21%		0.21%		0.00%		0.82%	
Health groups	Separated	30.91 ± 1.99	−10.47%	<0.001	9.31% ± 0.08%	7.55% ± 0.08%	1.55 ± 0.24	−2.67%	<0.001	−1.12%	<0.001	−2.98%	<0.001	−2.34%	<0.001	−0.02%	<0.001	−3.34%	<0.001
	Single	29.08 ± 1.45	−15.77%		30.00% ± 0.00%	7.71% ± 0.08%	1.63 ± 0.25	2.63%		1.11%		2.94%		2.31%		0.02%		3.29%	
Health surveillance	Absent	29.02 ± 1.87	−15.95%	<0.001	22.66% ± 0.09%	16.05% ± 0.07%	1.64 ± 0.26	2.90%	<0.001	1.25%	<0.001	3.48%	<0.001	4.44%	<0.001	0.03%	<0.001	3.49%	<0.001
	Continuous	30.88 ± 1.70	−10.57%		16.77% ± 0.14%	0.29% ± 0.01%	1.55 ± 0.23	−2.70%		−1.13%		−3.14%		−3.45%		−0.02%		−3.32%	
	Discontinuous	30.05 ± 1.87	−12.96%		19.81% ± 0.11%	6.74% ± 0.06%	1.59 ± 0.25	−0.14%		−0.09%		−0.27%		−0.90%		0.00%		−0.10%	
Milking stations	Suitable	30.12 ± 2.00	−12.77%	<0.001	19.73% ± 0.12%	7.63% ± 0.08%	1.57 ± 0.25	−0.97%	<0.001	−0.16%	<0.001	−0.42%	<0.001	−0.42%	0.32	0.00%	0.057	−1.66%	<0.001
	Not suitable	29.86 ± 1.92	−13.51%		19.73% ± 0.12%	7.63% ± 0.08%	1.61 ± 0.24	0.97%		0.16%		0.42%		0.42%		0.00%		1.66%	
Parlor maintenance	Suitable	30.12 ± 1.97	−12.76%	<0.001	19.42% ± 0.12%	7.07% ± 0.08%	1.58 ± 0.25	−0.64%	<0.001	−0.16%	<0.001	−0.44%	<0.001	−0.37%	0.33	0.00%	<0.001	−0.98%	<0.001
	Not suitable	29.86 ± 1.95	−13.52%		20.03% ± 0.12%	8.20% ± 0.08%	1.60 ± 0.25	0.64%		0.16%		0.44%		0.37%		0.00%		0.98%	
Milkers number	Very good	30.15 ± 1.95	−12.69%	<0.001	19.41% ± 0.12%	6.78% ± 0.08%	1.58 ± 0.25	−0.72%	<0.001	−0.20%	<0.001	−0.54%	<0.001	−0.44%	0.48	0.00%	<0.001	−1.08%	<0.001
	Good	30.06 ± 1.96	−12.94%		19.66% ± 0.12%	7.16% ± 0.08%	1.58 ± 0.25	−0.31%		−0.09%		−0.24%		−0.19%		0.00%		−0.46%	
	Insufficient	29.77 ± 1.97	−13.79%		20.11% ± 0.12%	8.96% ± 0.09%	1.61 ± 0.25	1.03%		0.28%		0.78%		0.63%		0.00%		1.55%	
Udder preparation	Suitable	30.31 ± 1.96	−12.23%	<0.001	19.18% ± 0.12%	6.41% ± 0.08%	1.56 ± 0.25	−1.70%	<0.001	−0.39%	<0.001	−1.07%	<0.001	−0.91%	0.02	0.00%	<0.001	−2.67%	<0.001
	Not suitable	29.68 ± 1.91	−14.05%		20.27% ± 0.11%	8.85% ± 0.09%	1.62 ± 0.24	1.70%		0.39%		1.07%		0.91%		0.00%		2.67%	
Post dipping	Suitable	30.48 ± 1.88	−11.73%	<0.001	16.99% ± 0.13%	4.75% ± 0.06%	1.57 ± 0.24	−1.49%	<0.001	−0.62%	<0.001	−1.71%	<0.001	−1.72%	<0.001	−0.01%	<0.001	−1.85%	<0.001
	Not suitable	29.50 ± 1.92	−14.56%		22.46% ± 0.09%	10.51% ± 0.09%	1.61 ± 0.25	1.49%		0.62%		1.71%		1.72%		0.01%		1.85%	
Mean		29.99 ± 1.96	−13.14%		19.73% ± 0.12%	7.63% ± 0.08%	1.59 ± 0.25												

^1^ FPCM: fat- and protein-corrected milk; ^2^ CM: contagious mastitis; ^3^ EM: environmental mastitis; ^4^ GWP: global warming potential.

**Table 2:** Effect of risk factors in automatic milking system scenario on milk yield, evaluated as protein- and fat-corrected milk (FPCM), incidence of contagious and environmental mastitis in the herd, and global warming potential (GWP; kg CO_2_eq/kg FPCM). The delta (∆) of GWP, enteric methane emissions, feeding consumption, bedding materials, energy consumption, and manure storage emissions were assessed in front of the mean value of all the scenarios.

Automatic Milking System (AMS)		FPCM ^1^	FPCM		CM ^2^ Herd	EM ^3^ Herd	GWP ^4^	GWP		Enteric Methane Emissions		Feed Consumption		Bedding Materials		Energy Consumption		Manure Storage	
		Scenario			Incidence	Incidence													
		Kg	∆	*p*- Value	%	%	kg CO_2_eq/kg FPCM	∆	*p*- Value	∆	*p*- Value	∆	*p*- Value	∆	*p*- Value	∆	*p*- Value	∆	*p*- Value
Resting area	Deep litter	34.56 ± 4.32	−0.57%	0.027	19.42% ± 0.12%	10.75% ± 0.11%	1.47 ± 0.29	2.88%	<0.001	0.21%	0.028	0.56%	0.046	49.47%	<0.001	0.00%	0.022	29.32%	<0.001
	Cubicle	34.95 ± 4.20	0.57%		19.31% ± 0.12%	8.55% ± 0.10%	1.39 ± 0.21	−2.88%		−0.21%		−0.56%		−49.47%		0.00%		16.65%	
Overcrowding	Absent	36.35 ± 4.20	4.60%	<0.001	18.25% ± 0.13%	8.13% ± 0.10%	1.31 ± 0.22	−8.74%	<0.001	−1.56%	<0.001	−4.34%	<0.001	−4.58%	0.135	−0.01%	<0.001	4.01%	<0.001
	Presence	33.16 ± 3.69	−4.60%		20.48% ± 0.11%	11.17% ± 0.11%	1.56 ± 0.24	8.74%		1.56%		4.34%		4.58%		0.01%		41.96%	
Resting area cleanliness	Clean	36.93 ± 4.06	6.27%	<0.001	16.94% ± 0.13%	4.02% ± 0.05%	1.31 ± 0.21	−8.08%	<0.001	−2.11%	<0.001	−5.70%	<0.001	−6.45%	0.037	−0.02%	<0.001	6.94%	<0.001
	Not clean	32.58 ± 3.21	−6.27%		21.79% ± 0.10%	15.28% ± 0.11%	1.55 ± 0.25	8.08%		2.11%		5.70%		6.45%		0.02%		39.03%	
Bedding material	Sand	35.24 ± 4.23	1.39%	0.048	19.37% ± 0.12%	6.84% ± 0.09%	1.23 ± 0.18	−13.89%	<0.001	−0.49%	0.048	−1.29%	0.058	−74.17%	<0.001	−0.01%	0.024	−11.01%	<0.001
	Straw. sawdust	34.98 ± 4.24	0.64%		19.37% ± 0.12%	7.90% ± 0.10%	1.32 ± 0.18	−7.44%		−0.23%		−0.65%		143.67%		0.00%		2.23%	
	Others	34.85 ± 4.30	0.29%		19.37% ± 0.12%	9.04% ± 0.10%	1.31 ± 0.19	−8.13%		−0.10%		−0.26%		130.48%		0.00%		0.28%	
	Composted manure-based materials	34.52 ± 4.24	−0.67%		19.37% ± 0.12%	10.90% ± 0.11%	1.59 ± 0.25	11.25%		0.23%		0.60%		−100.00%		0.00%		53.09%	
	Manure-based materials with straw	34.68 ± 4.29	−0.20%		19.37% ± 0.12%	10.25% ± 0.11%	1.52 ± 0.22	6.18%		0.07%		0.21%		0.03%		0.00%		38.86%	
	Not composted manure-based materials	34.26 ± 4.26	−1.44%		19.37% ± 0.12%	12.96% ± 0.12%	1.60 ± 0.25	12.03%		0.51%		1.39%		−100.00%		0.01%		54.46%	
Health groups	Separated	36.38 ± 4.99	4.68%	<0.001	8.73% ± 0.08%	9.65% ± 0.11%	1.39 ± 0.26	−3.15%	<0.001	−1.40%	<0.001	−3.63%	<0.001	−4.03%	0.461	−0.02%	<0.001	−73.92%	<0.001
	Single	33.13 ± 2.48	−4.68%		30.00% ± 0.00%	9.65% ± 0.11%	1.48 ± 0.25	3.15%		1.40%		3.63%		4.03%		0.02%		−73.18%	
Health surveillance	Absent	33.17 ± 2.75	−4.56%	<0.001	22.16% ± 0.10%	12.60% ± 0.11%	1.47 ± 0.25	3.08%	<0.001	1.39%	<0.001	3.39%	<0.001	4.07%	0.367	0.01%	<0.001	−73.19%	<0.001
	Continuous	37.28 ± 4.81	7.26%		16.57% ± 0.14%	3.74% ± 0.06%	1.36 ± 0.25	−5.01%		−2.25%		−5.47%		−6.51%		−0.02%		−74.15%	
	Discontinuous	33.82 ± 3.75	−2.70%		19.37% ± 0.12%	12.60% ± 0.11%	1.46 ± 0.26	1.93%		0.85%		2.08%		2.44%		0.01%		−73.33%	
AMS type	Type A	36.31 ± 4.13	4.47%	<0.001	17.14% ± 0.13%	2.72% ± 0.05%	1.38 ± 0.24	−3.57%	<0.001	−1.53%	<0.001	−4.04%	<0.001	−4.02%	0.691	−0.02%	<0.001	17.24%	0.004
	Type B	35.59 ± 4.23	2.41%		18.08% ± 0.13%	5.30% ± 0.06%	1.40 ± 0.25	−1.96%		−0.84%		−2.28%		−2.41%		−0.01%		19.88%	
	Type C	33.33 ± 3.91	−4.09%		21.50% ± 0.11%	16.56% ± 0.11%	1.48 ± 0.27	3.31%		1.41%		3.79%		3.87%		0.02%		28.28%	
	Type D	33.79 ± 4.07	−2.78%		20.75% ± 0.11%	14.01% ± 0.11%	1.46 ± 0.26	2.22%		0.95%		2.52%		2.57%		0.01%		26.54%	
Mean		34.75 ± 4.26	0.00%		19.37% ± 0.12%	9.65% ± 0.11%	1.43 ± 0.26												

^1^ FPCM: fat- and protein-corrected milk; ^2^ CM: contagious mastitis; ^3^ EM: environmental mastitis; ^4^ GWP: global warming potential.

## Data Availability

The original contributions presented in the study are included in the article/Supplementary Materials. Further inquiries can be directed to the corresponding author.

## Supplementary Materials

The following supporting information can be downloaded at: https://www.mdpi.com/article/10.3390/ani15010050/s1, Table S1: Parameters of the production model; Table S2: Weight of the component considered to assess the incidence of contagious and environmental mastitis risk in the herd; Table S3: Model variables of the two main model components: resting area and milking area; Table S4: Weight of each bedding material type on environmental mastitis risk; Table S5: Milking area parameters of milking parlor scenarios; Table S6: Global warming potential of the daily supply of bedding material into deep litter and cubicle scenarios. The values are reported as kg CO_2_eq/day.
