# Supplementary material for: Modeling Mastitis Risk Management Effects on Dairy Milk Yield and Global Warming Potential
Source: Animals (Basel). 2024 Dec 28;15(1):50. doi: 10.3390/ani15010050 (PMC11718989; doi:10.3390/ani15010050)
Supplement: Supplementary file 1 [file animals-15-00050-s001.zip › animals-3358502-supplementary.pdf]

Supplementary Materials

Modeling Mastitis Risk Management Effects on Dairy Milk Yield and Global Warming Potential

Giulia Ferronato [1,*], Anna Simonetto [1], Gianni Gilioli [1] and Alfonso Zecconi [2]

[1] Department of Civil Engineering, Architecture, Environment, Land Planning and Mathematics (DICATAM), University of Brescia, 25121 Brescia, Italy; anna.simonetto@unibs.it (A.S.); gianni.gilioli@unibs.it (G.G.)
[2] One Health Unit, Department of Biomedical, Surgical and Dental Sciences, School of Medicine, University of Milan, 20133 Milan, Italy; alfonso.zecconi@unimi.it
* Correspondence: giulia.ferronato@unibs.it

Table S1. Parameters of the mastitis-risk and milk production assessment model

Parameter	Description	Unit of measure	Value
α_C	The quantity of milk lost during a lactation period by an animal with contagious mastitis	proportion	0.25
α_E	The quantity of milk lost during a lactation period by an animal with environmental mastitis	proportion	0.25
β_{YP}	Milk yield increase due to Automatic Milking System	proportion	0.3
γ_{CM}	Maximum proportion of animals in a herd with contagious mastitis	proportion	0.3
δ_{EM}	Maximum proportion of animals in a herd with environmental mastitis	proportion	0.5
ρ_{RY}	Milk yield reduction due to overcrowding	proportion	0.1
ρ_{OC}	Weight of the component "Overcrowding" for RY_{RA}	Pure number	0.5: If deep deep litter<br>0.7: il cubicle
ρ_{DR}	Weight of the component "Cleanliness" for RY_{RA}	Pure number	0.5: If deep deep litter<br>0.3: il cubicle
γ_{OC}	Weight of the component "Overcrowding" forCM_{RA}	Pure number	0.5
γ_{DR}	Weight of the component "Cleanliness" for CM_{RA}	Pure number	0.5
δ_{OC}	Weight of the component "Overcrowding" for EM_{RA}	Pure number	0.25: if resting area = deep litter<br>0.15: if resting area = Cubicle
δ_{DR}	Weight of the component "Cleanliness" for EM_{RA}	Pure number	0.25: if resting area = deep litter<br>0.15: if resting area = Cubicle
I_{YP}	Coefficient of milk yield reduction in the presence of Automatic Milking System in proportion to the level of mastitis presence in the barn	Pure number	1.0: If CM=0 and $EM = 0$<br>0.5: If CM>0 and $EM = 0$<br>0.8: If CM=0 and $EM > 0$<br>0.3: If CM>0 and $EM > 0$
γ_{R1}. δ_{R1}	Weight of type 1 AMS for CM_{MA} or EM_{MA} (rispectively)		0.1: if resting area is clean<br>0.2: if resting area is dirty
γ_{R2}. δ_{R2}	Weight of type 2 AMS for CM_{MA} or EM_{MA} (rispectively)		0.2: if resting area is clean<br>0.4: if resting area is dirty
γ_{R3}. δ_{R3}	Weight of type 3 AMS for CM_{MA} or EM_{MA} (rispectively)		0.5: if resting area is clean<br>1.0: if resting area is dirty
γ_{R4}. δ_{R4}	Weight of type 4 AMS for al CM_{MA} or EM_{MA} (rispectively)		0.4: if resting area is clean<br>0.9: if resting area is dirty
ρ_{PN}	Impact coefficient of the resting area and post dipping on RY_{OM}	Pure number	0.4
ρ_{WN}	Impact coefficient of waiting area on RY_{OM}	Pure number	0.05
ρ_{MN}	Impact coefficient of milking stations number on RY_{OM}	Pure number	0.1

γ_{PN}	Impact coefficient of post-dipping on CM_{OM}	Pure number	1.0
δ_{PN}	Impact coefficient of milking stations number on RY_{OM}	Pure number	0.6
δ_{DR}	Impact coefficient of resting area on CM_{OM}	Pure number	0.4

Table S2. Weight of the component considered to assess the incidence of contagious and environmental mastitis risk in the herd

			AMS & deep deep litter	AMS & Cubicle	MP & deep deep litter	MP & Cubicle
ω_{RA}	Component weight of resting area on total contagious mastitis risk	Pure number	0.5	0.3	0.4	0.3
ω_{MA}	Component weight of milking management on total contagious mastitis risk	Pure number	0.5	0.7	0.1	0.3
ω_{OM}	Component weight of "Other milking characteristics" on total contagious mastitis risk	Pure number	0.0	0.0	0.5	0.4

Table S3. Model parameters of the resting area and milking area

I_{OC}	overcrowding of resting area	0: Absent<br>1: Overcrowding
I_{DR}	dirtiness resting area	0: Clean<br>1: Dirty
I_{PN}<br>I_{WN}<br>I_{MN}	Suitability of post-dipping<br>Suitability of waiting area<br>Suitability of milking units	0: Suitable<br>1: Not suitable
I_{MM}	Status of milk monitoring	0 = absent<br>0.2 = discontinuous<br>0.5 = continuous
I_{R1-R4}	Automatic Milking System type 1. type2. type 3. type 4	0: absent<br>1: present

Table S4. Weight of each bedding material type on environmental mastitis risk for the Automatic Milking System (AMS) scenarios

δ_{MT}	Sand	Straw	Other materials	Solid separated digestate with straw	Composted solid separated digestate	Not composted solid separated digestate
Deep litter	0	0.1	0.2	0.3	0.35	0.5
Cubicle	0	0.06	0.12	0.18	0.21	0.3

Table S5. Milking area parameters of milking parlour (MP) scenarios

Milkers number	Udder preparation	Maintenance	ρ_{MP}	γ_{MP}	δ_{MP}
Good	Suitable	Suitable	0.00	0.00	0.00
		Not suitable	0.05	0.40	0.25
	Not suitable	Suitable	0.10	0.60	0.50
		Not suitable	0.15	0.60	0.50
Discrete	Suitable	Suitable	0.00	0.00	0.00
		Not suitable	0.05	0.40	0.38
	Not suitable	Suitable	0.10	0.60	0.50
		Not suitable	0.20	1.00	0.63
Insufficient	Suitable	Suitable	0.05	0.40	0.38
		Not suitable	0.05	0.60	0.50
	Not suitable	Suitable	0.20	0.80	0.75
		Not suitable	0.20	1.00	1.00

Table S6. Global Warming Potential of the daily supply of bedding material into deep litter and cubicle scenarios. The Values are reported as kg CO_2eq/day.

λ_{MT}	Sand	Straw	Other materials	Solid separated digestate with straw	Composted solid separated digestate	Not composted solid separated digestate
Deep litter	0.07	2.21	2.08	0.41	0	0
Cubicle	0.11	0.82	0.86	0.41	0	0
